# Classifying the Role of Surface Ligands on the Passivation and Stability of Cs_2_NaInCl_6_ Double Perovskite Quantum Dots

**DOI:** 10.3390/nano14040376

**Published:** 2024-02-17

**Authors:** Keita Tosa, Chao Ding, Shikai Chen, Shuzi Hayase, Qing Shen

**Affiliations:** 1 Faculty of Informatics and Engineering, The University of Electro-Communications, 1-5-1 Chofugaoka, Chofu, Tokyo 182-8585, Japan; tosa@jupiter.pc.uec.ac.jp (K.T.); chen@jupiter.pc.uec.ac.jp (S.C.); hayase@uec.ac.jp (S.H.); 2 Institute of New Energy and Low-Carbon Technology, Sichuan University, Chengdu 610065, China

**Keywords:** Cs_2_NaInCl_6_, quantum dots, stability, ligands, oleylamine, oleic acid

## Abstract

Cs_2_NaInCl_6_ double perovskites, which have excellent photoelectric conversion properties and are non-toxic and lead-free, have recently gained significant attention. In particular, double-perovskite quantum dots (QDs) are viewed as a promising material for optoelectronic device applications. Ligands such as oleic acid (OA) and oleylamine (OAm) are essential for the synthesis of perovskite QDs, but their specific roles in double-perovskite QDs remain unclear. In this study, we have investigated the binding of OA and OAm to Cs_2_NaInCl_6_ QDs through FTIR and NMR and their effects on the surface defect reduction and stability improvement for Cs_2_NaInCl_6_ QDs. We found that only OAm was bound to the QD surfaces while OA was not. The OAm has a significant effect on the photoluminescence quantum yield (PLQY) improvement by passivating the QD surface defects. The stability of the QDs was also evaluated, and it was observed that OA played a significant role in the stability of the QDs. Our findings provide valuable insights into the roles of ligands in influencing the photophysical properties and stability of lead-free double-perovskite QDs.

## 1. Introduction

Lead (Pb) halide perovskites with the molecular formula ABX_3_ (A: CH_3_NH_3_, Cs, etc.; B: Pb; X: Cl, Br, and I) have attracted much attention because of their remarkable optoelectronic properties and potential applications such as solar cells, LED, and light sources for displays [1,2,3,4]. Among these, quantum dots (QDs) have been the focus of much research in recent years due to their simplicity of synthesis and ease of application to devices [2,5,6,7]. However, the toxicity and instability of Pb-based perovskites greatly hinder practical applications [8]. Thus, studies on Pb-free perovskites are necessary and important.

Pb^2+^ in perovskites can be substituted by tin (Sn^2+^) or germanium (Ge^2+^). However, these replacements often lead to instability due to the propensity of Sn^2+^ and Ge^2+^ to oxidize into Sn^4+^ and Ge^4+^, respectively [9,10]. Consequently, some research has recently shifted focus to double perovskites with A_2_B′(I)B″(III)X_6_, where Pb^2+^ is replaced by a combination of monovalent and trivalent cations [11,12]. Various double perovskite families have been investigated, including Cs_2_AgBiBr_3_, but they have a direct band gap problem, which is an optically forbidden transition [13]. This issue has been addressed through chemical doping, leading to demonstrations of high-efficiency photoluminescence (PL) [14]. The success of chemical doping is tied to the soft lattice characteristic of double perovskites, which facilitates strong electron–phonon coupling. Excitation triggers the Jahn–Teller effect, giving rise to self-trapped excitons (STEs) [15].

**Figure 1:**
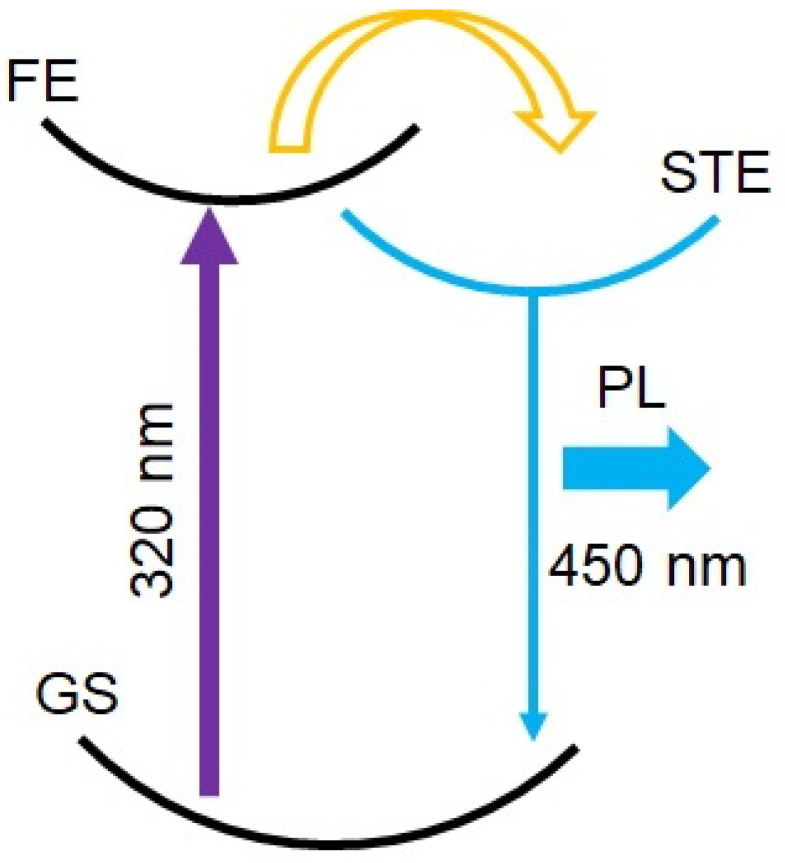
Schematic diagram of the STE.

Double perovskites that generate white light have been mainly studied, but double perovskites that emit blue light have rarely been reported. Recently, Cs_2_NaInCl_6_ with A: Cs^+^, B′(I): Na^+^, B″(III): In^3+^, and X: Cl^−^ has been proposed and studied as a promising material for solid-state lighting [16,17,18,19,20]. This double perovskite also has a direct band gap with forbidden transitions, and low PL has been an issue; in 2020, Cs_2_NaInCl_6_ doped with Sb^3+^ broke the parity forbidden condition and went from a dark self-trapped exciton (STE) to a bright STE, resulting in a significant improvement in PL quantum yield (PLQY) of blue emission [14]. Here, the STE is briefly explained (Figure 1). When nothing is doped, it has a direct band gap structure, which is a forbidden transition (FE→GS transition). When Sb^3+^ is doped, a new absorption band is generated, and a new emission band, the STE level, is also generated. This breaks the forbidden transition and thus can emit light (STE→GS).

To apply perovskite QDs to LED devices, it is necessary to improve the PLQY and stability of the solution for storage. A major factor influencing the PLQY of Cs_2_NaInCl_6_ double-perovskite QDs are Sb^3+^ doping and surface ligands—Sb^3+^ is thought to substitute In^3+^. We synthesized and characterized the physical properties of Sb^3+^ ligands with different Sb/(In + Sb) ratios according to the report by Zeng et al. [17]. As a result, we found that the PLQY varied greatly with the amount of Sb^3+^ doping; the highest PLQY was obtained, when 10% Sb^3+^ was doped (as shown in Section 3.1 of this paper). When the doping level was increased to 40%, the PLQY decreased to 10%. When the Sb^3+^ doping was reduced to 5%, PLQY decreased to about 30%. Thus, the amount of Sb^3+^ doping had a significant effect on the photophysical properties of Cs_2_NaInCl_6_ double-perovskite QDs. Another factor, the surface ligand, is due to the fact that the Cs_2_NaInCl_6_ double perovskite is a “quantum dot”. Quantum dots are nanocrystals, with typically 5 to 20 nm in size, and are strongly affected by the surface due to their large surface-to-volume ratio. These quantum dots are covered with organic ligands in order to exist as colloids in solutions. In general, both oleic acid (OA) and oleylamine (OAm) are used in the synthesis of QDs. They act to dissolve the precursor in the high-boiling solvent used for synthesis, and these bind to the surface of QDs and function as surface ligands, enabling QDs to exist stably as a colloidal solution. Surface ligands are used to passivate the surface defect of QDs and prevent them from coalescing with each other [21], and it is known that the stability can be improved by appropriately selecting them [22]. Much research has been devoted to the binding of ligands to QDs and the effects of ligands on the physical properties of QDs [23,24,25,26]. Elucidating the properties of the surface ligands is necessary to synthesize QDs with high stability and high PLQY. In this study, we investigated the effects of two kinds of ligands, i.e., OA and OAm, on the optical properties and the stability of Cs_2_NaInCl_6_ double-perovskite QDs by adjusting the ratio of OA to OAm used during the QD synthesis. Fourier-transform infrared spectroscopy (FTIR) and nuclear magnetic resonance (NMR) were used to characterize the ligands states. The changes in various properties with the ratio of the two ligands were evaluated by measuring the PLQY, absorption and PL spectra, X-ray spectroscopy (XPS), X-ray diffraction patterns (XRD), and transmission electron microscopy (TEM) images. The roles of OAm and OA in the reduction of surface defects and improvement of the stability have been clarified.

## 2. Materials and Methods

### 2.1. Materials

Cesium acetate (Cs(OAc), 99.99%; Sigma-Aldrich, Tokyo, Japan), sodium acetate (Na(OAc), 99.995%; Sigma-Aldrich, Tokyo, Japan), indium acetate (In(OAc)_3_, 99.99%; Sigma-Aldrich, Tokyo, Japan), antimony acetate (Sb(OAc)_3_, 99.99%; Sigma-Aldrich, Tokyo, Japan), germanium (IV) chloride (GeCl_4_, 99.99%; FUJIFILM Wako Chemicals, Osaka, Japan), 1-octadecene (ODE, 90%; Sigma-Aldrich, Tokyo, Japan), oleic acid (OA, 90%; Sigma-Aldrich, Tokyo, Japan), oleylamine (OAm, 70%; Sigma-Aldrich, Tokyo, Japan), chlorobenzene (C_6_H_5_Cl, 99.0%; FUJIFILM Wako Chemicals, Osaka, Japan), hexane (C_6_H_14_, 96.0%; FUJIFILM Wako Chemicals, Osaka, Japan) were used as received without further purification.

### 2.2. Synthesis of Cs_2_NaInCl_6_ QDs

Cs_2_NaInCl_6_ double-perovskite QDs were synthesized by a modified method developed by Wang et al. [13]. Quantum dots were synthesized in air. Cs(OAc) (0.71 mmol), Na(OAc) (0.5 mmol), In(OAc)_3_ (0.495 mmol), and Sb(OAc)_3_ (0.055 mmol) in octadecene(ODE) (9 mL) were placed in a three-neck flask. Then, OA and OAm were placed in a three-necked flask with different ratios, i.e., the [OA]/[OAm] ratios were 4, 2, 1, 0.5, and 0.25, respectively, where the total volume of [OA] + [OAm] was 3.5 mL. The mixture was heated to 110 °C, stirred under vacuum for 50 min and then heated to 170 °C under a nitrogen atmosphere, and a GeCl_4_ precursor solution containing 77 μL of GeCl_4_ per 1 mL of ODE was swiftly injected. The solution was then heated to 180 °C. After 5 min, the reaction mixture was rapidly cooled in ice water to terminate the reaction. The reaction mixture was centrifuged at 9500 rpm for 5 min, and the precipitate was collected. The precipitate was mixed with 10 mL of chlorobenzene and centrifuged at 9500 rpm for 5 min, and the precipitate was collected. The precipitate was thoroughly dried, dispersed in 4 mL of hexane centrifuged at 4000 rpm for 5 min, and the supernatant containing the target quantum dots were collected.

### 2.3. Characterization

#### 2.3.1. X-ray Diffraction Pattern

Powder XRD data were obtained from QDs drop-cast on glass substrates using Rigaku SmartLab; the X-ray generator produced Cu Kα radiation (λ = 1.541 Å). Samples were scanned while rotating at 5 °s ^−1^. Background processing and particle size calculations were performed using Rigaku SmartLab II software (v4.5.286.0). 

#### 2.3.2. Transmission Electron Microscopy (TEM)

A JEOL 2100F field emission TEM operating at 200 kV was used to capture high-resolution TEM images. TEM samples were prepared by drop-casting a dilute dispersion of QDs in hexane onto a TEM grid.

#### 2.3.3. Optical Measurements

UV−visible absorbance spectra were measured using a Japan Spectroscopic V-760 spectrophotometer; PL emission spectra and PLQY were obtained using a HAMAMATSU Quantaurus-QY fluorescence spectrometer. PLE was measured using a Japan Spectroscopy FP-6500 spectrophotometer. For all optical measurements, QDs were dispersed in hexane in a quartz cuvette with a 1 cm optical path length.

#### 2.3.4. Fourier-Transform Infrared Spectroscopy

FTIR spectra were acquired using a Thermo Scientific Nicolet 6700. The accessory used was a Smart iTX single-reflection ATR accessory.

#### 2.3.5. Nuclear Magnetic Resonance Spectroscopy and Nuclear Overhauser Effect Spectroscopy

^1^HNMR and nuclear Overhauser effect (NOESY) measurements were performed using a JNM-ECZL-500R spectrometer operating at 500 MHz (11.74 T). QDs were dispersed in chloroform-d at a concentration of 10 mg/mL. ^1^HNMR data were acquired using a relaxation delay of 2 s and 64 scans; NOESY was collected using 8 scans with a spectral width of 15 ppm and a mixing time of 800 ms. ^1^HNMR and NOESY spectra were processed using JEOL Delta v6.2 software and calibrated with the chloroform peak present in chloroform-d.

## 3. Results and Discussion

### 3.1. Properties of Cs_2_NaInCl_6_ Double-Perovskite QDs

**Figure 2:**
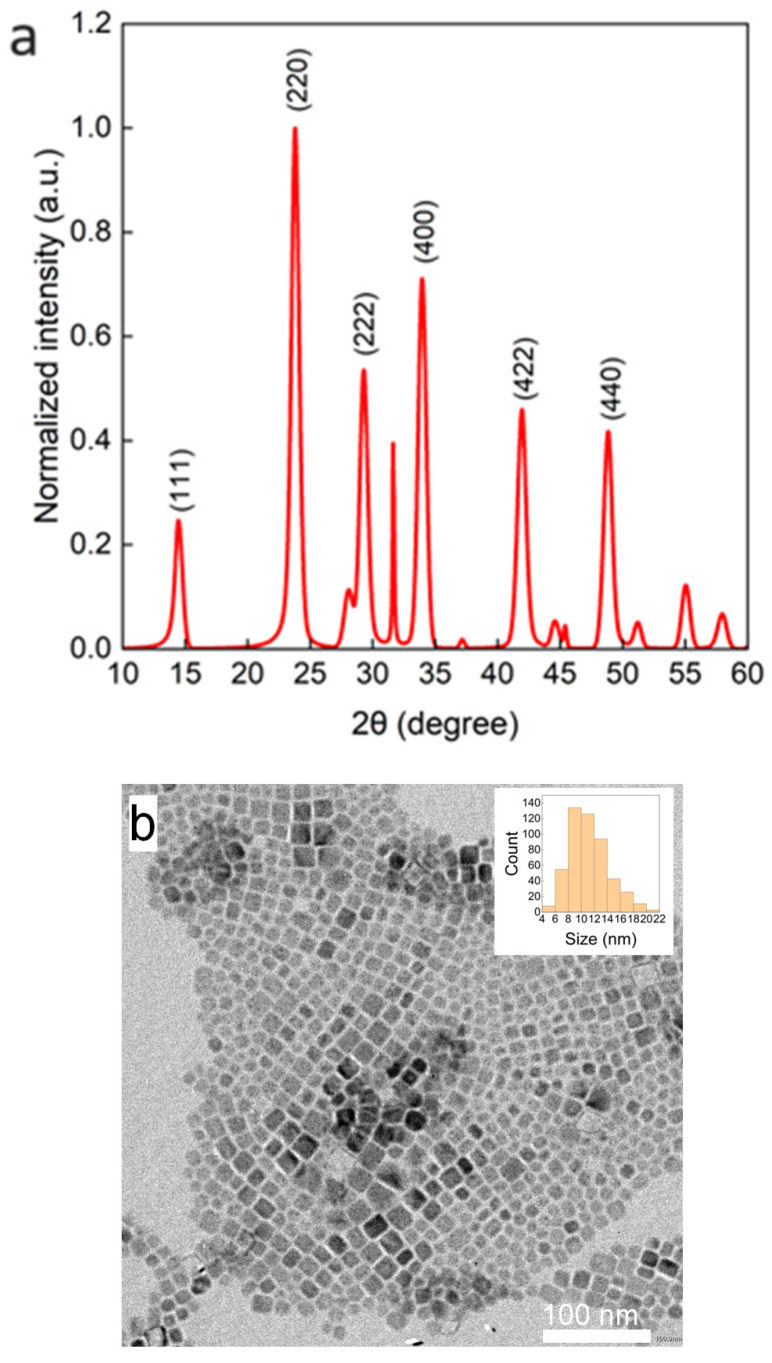
XRD pattern (**a**) and TEM image (**b**) of QDs synthesized with the condition of [OA]/[OAm] = 4.

The morphology and the crystal structure of the synthesized QDs under the condition of [OA]/[OAm] = 4 were characterized by the TEM image and the XRD pattern (Figure 2). The QDs showed a cubic shape. The average size of the QDs obtained from the TEM images was 11.2 nm, and the size estimated from the XRD diffraction pattern was 11.6 nm using the Scherrer equation. The XRD pattern is consistent with that of Cs_2_NaInCl_6_ [14], indicating that Cs_2_NaInCl_6_ QDs have been synthesized successfully.

**Figure 3:**
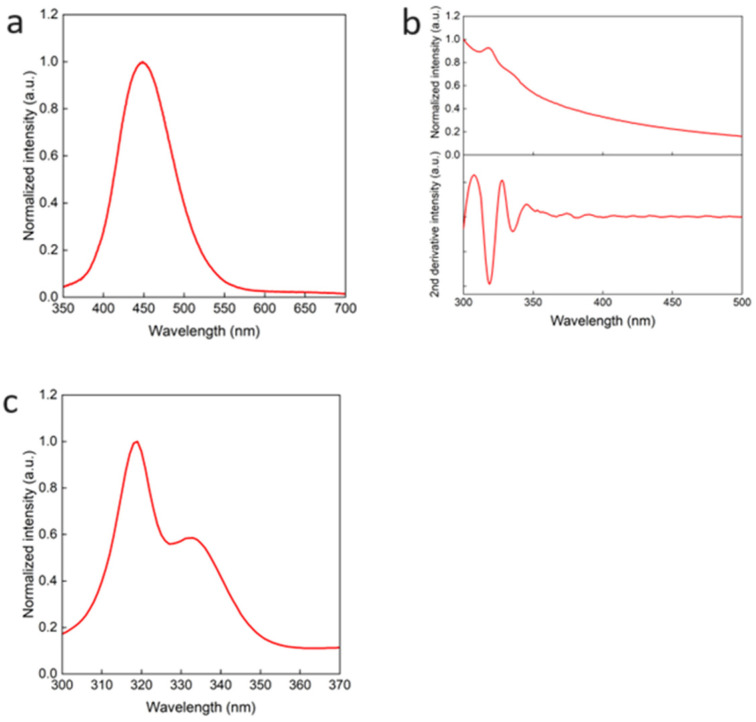
(**a**) PL spectrum obtained with an excitation wavelength of 320 nm; (**b**) optical absorption spectrum (**top**) and its second-derivative spectrum (**bottom**); and (**c**) PL excitation (PLE) spectrum of Cs_2_NaInCl_6_ QDs synthesized with the condition of [OA]/[OAm] = 4.

Figure 3 shows the typical PL, optical absorption, and PL excitation (PLE) spectra of the synthesized QDs under the condition of [OA]/[OAm] = 4. From the PL spectrum, a blue emission with a peak wavelength of 450 nm can be observed clearly. The PLQY of the luminescence was about 85%. In Figure 3b, besides the optical absorption spectrum (top), its second derivative spectrum (bottom) is also shown. The second-order differentiation of the absorption spectrum can be very useful for analyzing the spectrum of which the peaks are difficult to see, because it is known that the minima of the second-derivative spectrum corresponds to the peaks in the optical absorption spectrum [27]. Therefore, two absorption peaks at 320 nm and 335 nm can be observed clearly. These peaks were obtained by doping antimony (Sb), and when combined with the PLE (Figure 3c) peaks, the emission was strong at these absorption peaks. This broad blue luminescence has been reported to originate from the STE in the Cs_2_NaInCl_6_ QDs [17].

### 3.2. OA and OAm States in Cs_2_NaInCl_6_ Double-Perovskite QDs

Lead halide perovskite QDs have been the most studied among perovskite QDs, and especially CsPbBr_3_ perovskite QDs have been the subject of much interest. CsPbBr_3_ has also been studied, because it is a type of QDs and is strongly influenced by the surface and the ligand [22,24,28,29]. NMR has been used in these studies, and NMR has traditionally been used to identify organic compounds. Since CsPbBr_3_ is all-inorganic, NMR can be used to measure only organic ligands. There are two types of NMR reported: ^1^H NMR, which measures only protons, and nuclear Overhauser effect spectroscopy (NOESY), which uses the nuclear Overhauser effect (NOE). ^1^HNMR measurements show that when the ligand is bound to the quantum dot, the chemical shift peak shifts and the spectrum broadens due to the slow molecular tumbling effect and short T_2_ relaxation time. NOESY is a 2D measurement and is usually used to determine proton distance information, but in the case of ligands, it takes advantage of the fact that the sign of the peak changes between the bound and free species. The ligand bound to QDs slows down the molecular motion and behaves like a macromolecule. This causes the NOE signal to be negative, which is the same as the diagonal peak. On the other hand, the free ligand behaves like a small molecule, which shows a positive NOE signal (opposite to the diagonal peak, usually plotted in a different color from the diagonal peak).

**Figure 4:**
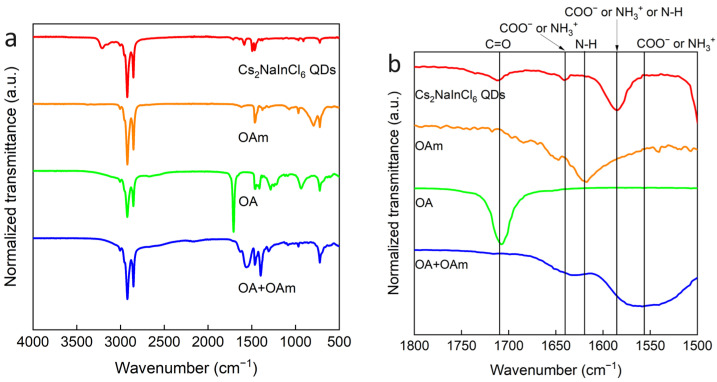
(**a**) FTIR spectra of Cs_2_NaInCl_6_ QDs synthesized with [OA]/[OAm] = 4, OAm, OA, and a mixture of OAm and OA. (**b**) Enlarged region of (**a**) where characteristic peaks for OA and OAm can be observed clearly.

OA and OAm are used in the synthesis of Ds, which are expected to bind to QDs produced. To confirm this expectation, FTIR and NMR measurements were carried out. Many studies have evaluated surface ligands using NMR alone, but it is also important to know whether ligands are ionized or not in order to study their behavior in solutions. Therefore, we investigated ligands present in solutions using FTIR. Figure 4 shows the FTIR spectra of four kinds samples: Cs_2_NaInCl_6_ QDs, OAm, OA, and a mixture of OAm and OA. The characteristic peak of OA was obtained at 1710 cm^−1^. This peak was also observed in the QD solution. The broad peaks at 1640 cm^−1^ and 1555 cm^−1^ were thought to be the peaks of deprotonated OA and protonated OAm, which are also partly contained in the QD solution. Finally, the peak at 1585 cm^−1^, which was only observed in the QD solution, was considered to be a shifted peak of ionized OA or ionized OAm. These results indicate that the QD solution contained OA, deprotonated OA, and protonated OAm.

**Figure 5:**
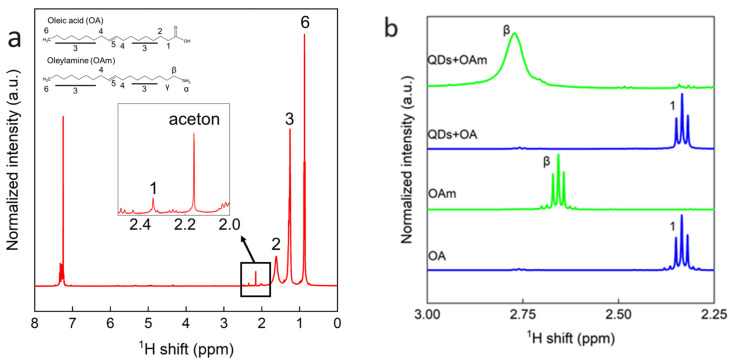
(**a**) NMR spectrum of Cs_2_NaInCl_6_ QDs synthesized with the condition of [OA]/[OAm] = 4. (**b**) NMR spectrum with the enlarged region where characteristic peaks for OA and OAm can be observed clearly. Measurements were made for QDs solutions with an addition of 10 μL of OA and OAm, respectively, and pure OA and OAm.

However, it is impossible to determine whether the two kinds of ligands are bound to the QDs or not only by the FTIR results. Therefore, NMR measurements were performed. Figure 5a shows the results of ^1^HNMR. In the QDs solution, the three and six protons common to OA and OAm, as well as the one and two peaks characteristic of OA, were observed. The sharp peak seen around 2.17 ppm was acetone used to clean the sample tube [30]. The amine peak of OAm could not be observed, because the amount of OAm in the solution was small or because it was close to the surface [31]. Therefore, we dropped 10 μL of OA and OAm into the QD solution and measured them (Figure 5b). In the sample with OA, the OA peak at 2.33 ppm was enhanced, but the peak did not shift, and no broadening of the peak was observed. This indicates that there was no interaction between OA ligand and the QDs, which suggest that OA was not bound to the QDs. On the other hand, the β peak at 2.66 ppm was shifted in the sample with an addition of OAm. This is because the OAm binding to the QDs restricts the longitudinal relaxation, resulting in a broadening of the peak due to the predominance of transverse relaxation [32,33]. These results indicate that there was an interaction between OAm ligand and the QDs.

**Figure 6:**
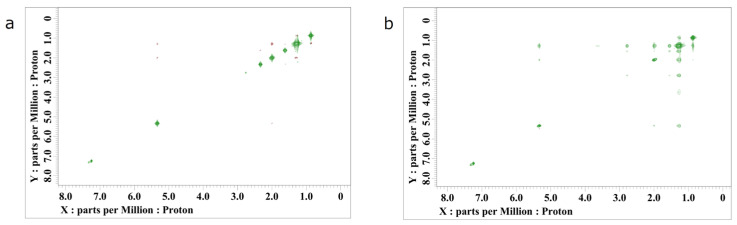
NOESY spectra of Cs_2_NaInCl_6_ QDs synthesized with the condition of [OA]/[OAm] = 4, in which 10 μL of OA was added (**a**) or 10 μL of OAm was added (**b**). Green is a negative NOE signal and red is a positive NOE signal.

Nuclear Overhauser spectroscopy (NOESY) was further performed to provide evidence that OA was not bound to the QDs but OAm was bound (Figure 6). First, nothing could be measured, when only the QD solution was used. This is thought to be due to the same reason as for ^1^HNMR. Next, we measured the QD solution with OA or OAm added, as in ^1^HNMR measurements. Red contour lines were observed in the sample with OA addition. A positive NOE signal was generated, which is the evidence that the QDs were not bound with OA. Next, a negative NOE signal was observed in the sample to which OAm was added. This is the evidence that the OAm was bound to the QDs. This is because the unbound ligand behaved as a small molecule, while the bound ligand behaved as a large molecule [34].

### 3.3. Roles of OA and OAm in Cs_2_NaInCl_6_ QDs

Based on the above results, it was shown that only OAm was bound to the QDs. Next, it is important and necessary to elucidate the respective roles of OA and OAm on the photophysical properties such as PLQY and the stability. Therefore, we prepared five kinds of QDs by changing the ratio of [OA] and [OAm] in the synthesis process, i.e., the [OA]/[OAm] ratios were 4, 2, 1, 0.5, and 0.25. We attempted to synthesize [OA]/[OAm] = 8 with an excess of OA and an OA-only sample. However, in both cases, quantum dots showing strong PL were not synthesized, and a few emission wavelengths that were observed were blue-shifted. This suggests that not only OA but also a certain amount of OAm is required for the synthesis of quantum dots. Conversely, when synthesis was attempted using only OAm, the acetic acid compound was almost insoluble in ODE, a high-boiling solvent, making the synthesis itself difficult. This clearly indicates that OA is indispensable for the synthesis and has the effect of dissolving acetic acid compounds as shown below, where (OOCR) indicates oleic acid and (RCH_2_NH_3_) indicates oleylamine:

(1) $$Cs\left(OAc\right)\to Cs\left(OOCR\right)+\left(RCH_{2}NH_{3}\right)\left(OAc\right)$$

**Figure 7:**
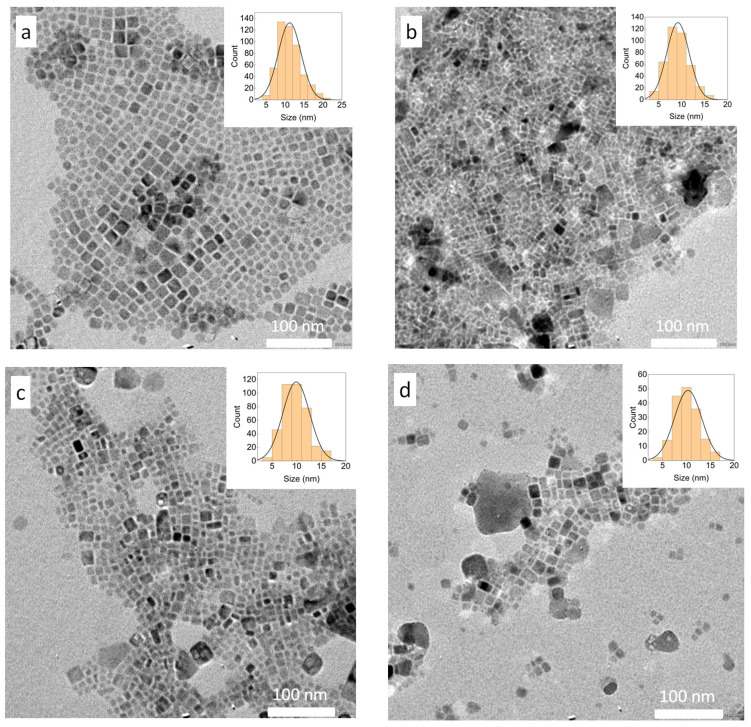

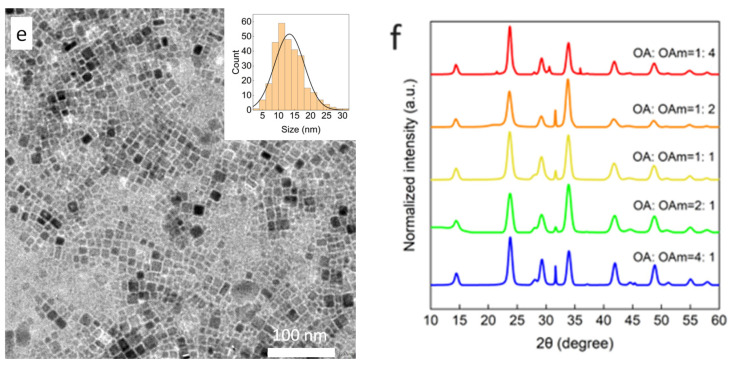
TEM images of Cs_2_NaInCl_6_ QDs synthesized with [OA]/[OAm] = 4 (**a**), [OA]/[OAm] = 2 (**b**), [OA]/[OAm] = 1 (**c**), [OA]/[OAm] = 0.5 (**d**), and [OA]/[OAm] = 0.25 (**e**). (**f**) XRD patterns of the five kinds of QDs synthesis with different [OA]/[OAm] ratios.

The TEM images and the XRD patterns of the five kinds of QDs shown in Figure 6. Until now, many studies have been reported on the synthesis of organic ligands with different carbon chain lengths and ratios of alkyl carboxylic acids to alkylamine ligands such as OA and OAm. Among them, it has been reported that they can become nanowires [35], nanosheets [36], nanospheres [37], nanocubes [35], hexagons, and heptagons [38]. Thus, the influence of ligands on QDs is not only limited to optical properties, but also has a significant effect on the shape. Therefore, we first investigated whether the shape of QDs changes when [OA]/[OAm] is changed. However, the synthesized QDs generally showed cubic shapes (Figure 7a–e). In addition, XRD results showed that the crystal structures of the QDs were cubic for all kinds of samples (Figure 7f), and the average sizes of the QDs obtained from the TEM images were 11.2 ± 3.0, 9.2 ± 2.4, 9.9 ± 2.7, 10.3 ± 2.8, and 13.4 ± 4.6 nm when the ratios of [OA]/[OAm] were 4, 2, 1, 0.5, and 0.25, respectively. Particle size measurements from TEM images were taken from wide-area images at a 40,000× magnification.

**Figure 8:**
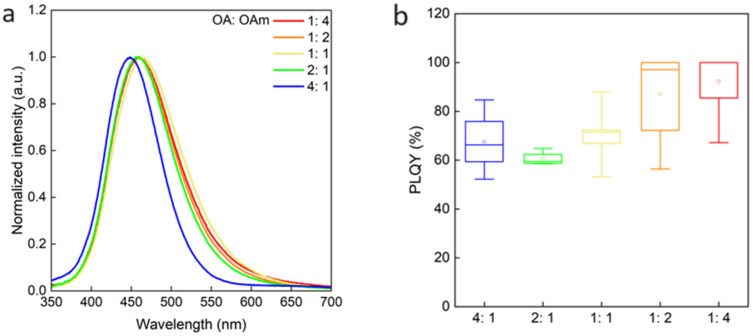
PL spectra (**a**) and PLQYs (**b**) of Cs_2_NaInCl_6_ QDs synthesized with different [OA]/[OAm] ratios.

Figure 8a,b show the PL spectra and the PLQYs of the five kinds of QDs. Figure 7b shows a box-and-whisker plot of the composites for each condition, synthesized six to eight times. The point near the center of the box represents the average value. As the [OA]/[OAm] changed, the PL peak wavelength was almost the same, and the shift of a few nm is possibly due to a little concentration change of the QD solution. On the other hand, the PLQY increased, as [OA]/[OAm] was decreased, i.e., as the ratio of OAm increased as shown in Figure 8b. In addition, the PLQY could be achieved close to 100%, when the [OA]/[OAm] ratios were 0.5 and 0.25. The high PLQY is thought to be due to the passivation of surface defects of the QDs when the OAm ratio was increased in the synthesis. From the previous result that only OAm was bound to the QDs, it is clear that OAm ligand plays a role in passivating the surface defects of the Cs_2_NaInCl_6_ QDs.

**Figure 9:**
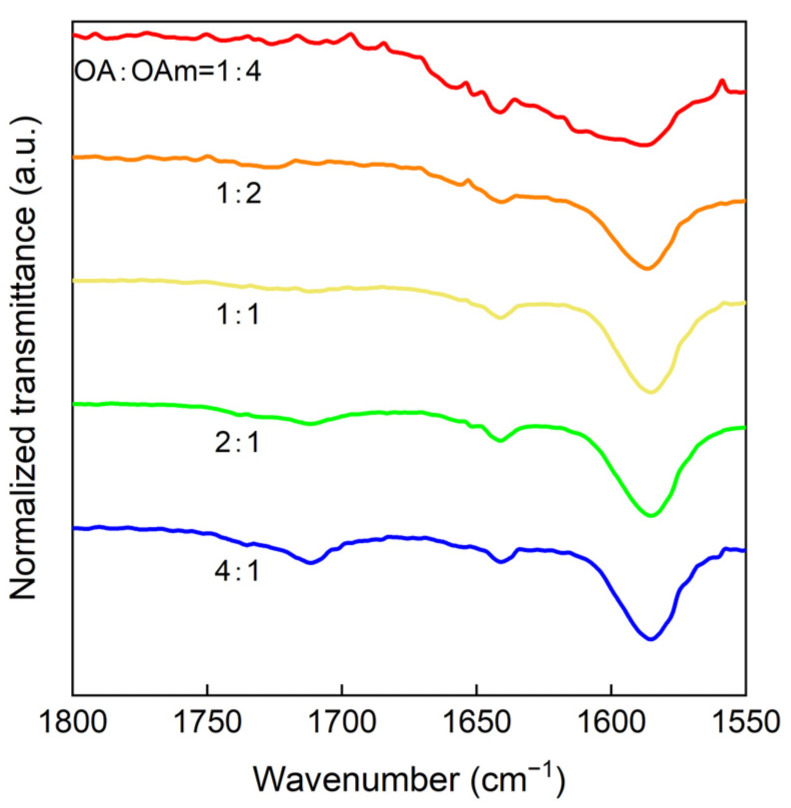
FTIR spectra of Cs_2_NaInCl_6_ quantum dots synthesized by changing the [OA]/[OAm] ratio.

FTIR measurement was performed to investigate the OA and the OAm in the QD solution for different [OA]/[OAm] ratios (Figure 9). As a result, the C=O peak at 1710 cm^−1^, which is a characteristic of OA, became smaller as [OA]/[OAm] decreased, and no peak was observed when the [OA]/[OAm] ratios were 0.5 and 0.25. This indicates that there was little residual OA in the QD solution.

**Figure 10:**
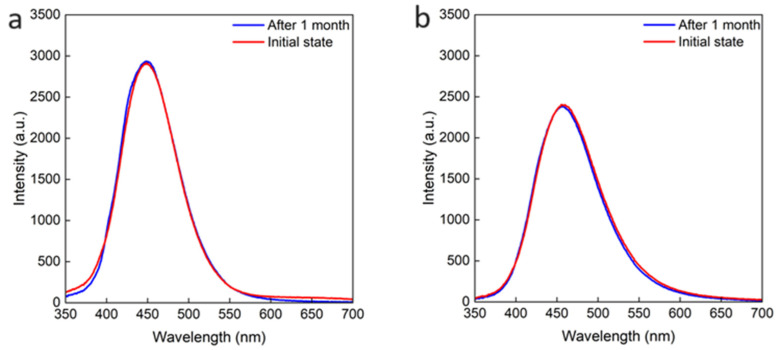

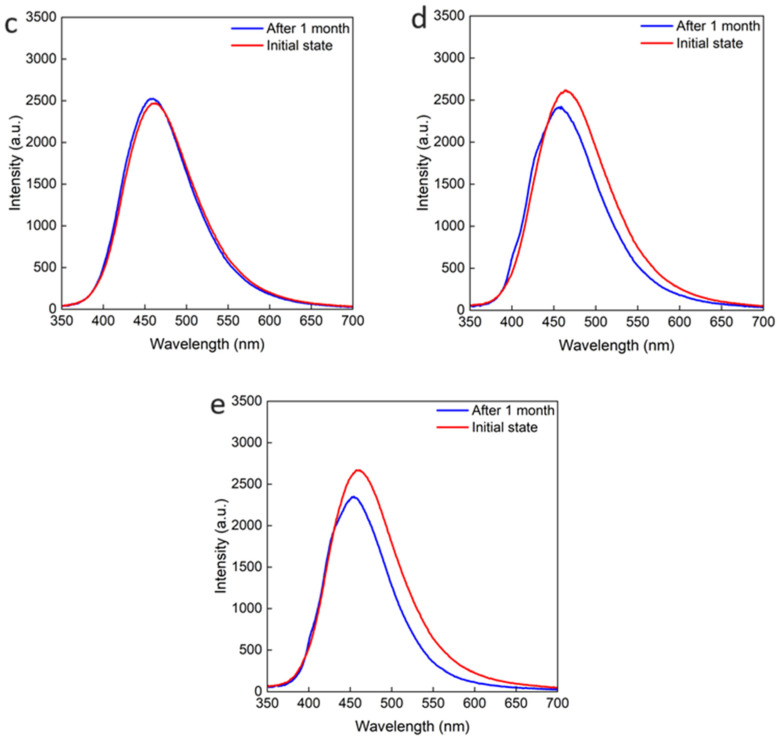
PL spectra of Cs_2_NaInCl_6_ QDs synthesized at [OA]/[OAm] = 4 (**a**), [OA]/[OAm] = 2 (**b**), [OA]/[OAm] = 1 (**c**), [OA]/[OAm] = 0.5 (**d**), and [OA]/[OAm] = 0.25 (**e**), which were measured soon after preparation and stored at room temperature and in air for 1 month.

Next, to investigate the change in stability when the [OA]/[OAm] ratio changes, we measured and compared the PL spectra of the five kinds of samples after one month of storage in the laboratory at room temperature and under air (Figure 10a–e). For the samples with the [OA]/[OAm] ratios of 4, 2, and 1, the PL spectra and the PL intensity were not changed after one month, indicating that the QDs were not degraded. On the other hand, for the [OA]/[OAm] ratios of 0.5 and 0.25, the PL intensity decreased, and the PL spectrum also changed after one month, indicating that the stability of the QDs became worse when the [OA]/[OAm] ratio was decreased. In summary, the samples with high OA content showed high stability. This is because OA could ionize OAm and the latter was then reattached to the QDs after they left the QDs. In samples where the QD solution did not contain enough OA, it is thought that the OA could not reionize the released OAm. We compared the stability in terms of the PLQY as well as the PL intensity, which was evaluated in terms of photons emitted relative to photons absorbed. However, we could not obtain accurate PLQY values because of the decrease in absorbance at 320 nm for the [OA]/[OAm] ratios of 0.5 and 0.25, where the PL intensity was reduced. The decrease in absorbance may be due to the decrease in the proportion of Sb-doped QDs. In other words, the proportion of QDs that can aggregate and absorb light has decreased due to the removal of the quantum dot ligands, as can be seen in the PL results, where the proportion of luminescent QDs has decreased.

**Figure 11:**
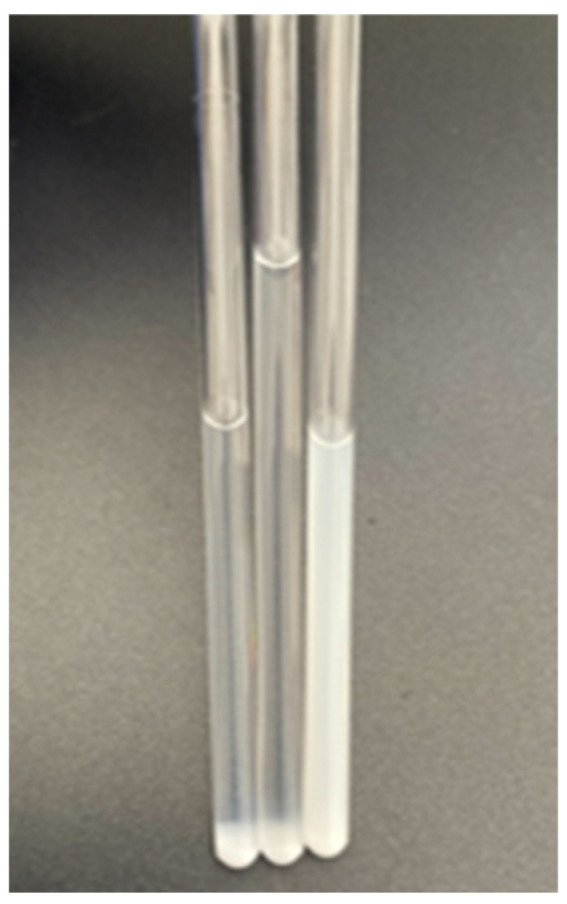
Aging in the ligand-rich state of the QD solutions prepared with [OA]/[OAm] = 0.25. From left to right: QDs solution; QD solution with OAm; QD solution with OA.

To confirm that OA ionized OAm and bound itself back to the QDs, we compared the stability of a sample of QD solution with a [OA]/[OAm] ratio of 0.25 and a sample added with OAm when stored at room temperature and in air for 72 h (Figure 11). When the surface ligands of the QDs were removed, the QDs aggregated and precipitated. This can be confirmed visually, indicating that a solution without precipitation was highly stable. The QDs precipitated in the pure QD and OAm-added samples. On the other hand, no precipitation was observed in the sample to which oleic acid was added. This indicates that OA ionized OAm and bound itself to the QDs, as expected above.

## 4. Conclusions

To elucidate the states of OA and OAm ligands in the Cs_2_NaInCl_6_ QDs solution synthesized with [OA]/[OAm] = 4, FTIR and NMR measurements were performed. The FTIR measurements revealed the presence of OA, deprotonated OA, and protonated OAm in the QD solution. Next, NMR measurements showed that OA and the QDs did not interact and were not bound to each other. On the other hand, OAm and QDs interacted with each other, indicating that they were bound. The results of synthesizing the QDs with different OA/OAm ratios showed that OAm contributed to the PLQY enhancement by passivating the surface defects of the QDs. On the other hand, OA was found to contribute to high stability by protonating OAm. Our findings offer critical insights into the roles of OA and OAm for photophysical properties and stability of lead-free double-perovskite QDs and lays a foundation for future research and technological advancements in this domain. In addition, this is considered to be significant progress toward LED device applications.

## Data Availability

The data that support the findings of this study are available from the corresponding author upon reasonable request.
